# Development of a measurement tool to assess local public health implementation climate and capacity for equity-oriented practice: Application to obesity prevention in a local public health system

**DOI:** 10.1371/journal.pone.0237380

**Published:** 2020-09-28

**Authors:** Katherine A. Stamatakis, Elizabeth A. Baker, Allese McVay, Hannah Keedy

**Affiliations:** 1 Department of Epidemiology and Biostatistics, Saint Louis University College for Public Health & Social Justice, St. Louis, Missouri, United States of America; 2 Department of Behavioral Science and Health Education, Saint Louis University College for Public Health & Social Justice, St. Louis, Missouri, United States of America; 3 Center for Innovation in Pediatric Practice (The Abigail Wexner Research Institute), Nationwide Children's Hospital, Columbus, Ohio, United States of America; European Monitoring Centre for Drugs and Drug Addiction, PORTUGAL

## Abstract

**Objective:**

The objective of this study was to develop a measurement tool to capture local public health department (LHD) organizational characteristics that align with implementation of equity-oriented practice, which may be used to gauge progress in building public health structures and functions that address the needs of vulnerable populations and reduce health inequities.

**Methods:**

We developed and tested a measurement tool, with practitioner input, based on an implementation science framework and informed by previous work defining public health essential services and practice recommendations for health equity. Measures assessed types of vulnerable populations served by the LHD, organizational climate, and four equity-oriented practice areas, including: assessment and planning, monitoring and analysis, leadership support, and obesity prevention. We also assessed opportunities for capacity building by identifying training needs of practitioners. Primary data were collected from Missouri local health department practitioners (n = 92, 80% response rate) via an online questionnaire, with a subset of the sample providing data for test-retest reliability.

**Results:**

Measures of equity-oriented implementation climate indicated areas of variability with respect to strengths and gaps across LHDs. While implementation climate was strong with respect to perceived importance (86%), a substantial proportion of LHDs cited concern over other priorities conflicting with equity-oriented implementation (32%). Likewise, a strong internal push (67%) was often accompanied by limited external political (25%) and community support (40%). Implementation climate measures generally had good to excellent reliability and were significantly associated with areas of equity-oriented practice. Frequently identified (>70%) training needs included improving skills in applying frameworks, assessment methods, and evaluating collaborations around equity.

**Conclusion:**

We developed a theory-based, practitioner-informed questionnaire to assess capacity for equity-oriented practice and identify opportunities for capacity building in local public health departments to engage in effective change toward health equity.

## Introduction

The inequitable distribution of social, economic and structural determinants of health and system-wide gaps in addressing the health needs of vulnerable populations have led to stark disparities in morbidity and mortality [1–6]. In order to improve health equity, emphasis must be placed on creating programs, policies, and structures that address the social determinants that influence access to health promoting resources as well as behaviors and exposures to health risks [7, 8]. A support structure that addresses the specific needs of local communities is needed in order to implement effective equity-oriented practices.

Current efforts to set standards of practice across the US public health system may help align local public health practice with efforts to better address social determinants of health inequities. For example, the national public health accreditation program prescribes a number of standards and measures that address health equity [9]. Healthy People 2020 has numerous objectives and recommended interventions that address the social determinants of health [10]. It is, however, unclear to what extent local public health agencies currently have the capacities to align assessment, assurance and policy development to address equity and social determinants [11]. Tracking the unique aspects of organizational supports and workforce capacities required for equity-oriented practice is needed in order to strategically implement interventions to meet the needs of vulnerable populations in the community [12].

Recent work in implementation science offers a theoretical basis for understanding elements of organizational capacity for innovation, providing an important synthesis of models and key domains for identifying implementation-related constructs that can be translated into measurable standards of equity-oriented practice. The consolidated framework for implementation science (CFIR) is a widely used meta-framework that organizes numerous constructs across domains representing multiple levels both internal and external to the organizational environment [13]. Furthermore, the CFIR provides a foundation for operationalizing constructs, such as implementation climate, which may be amenable to organizational improvement strategies, into measures that can be repeated over time and across various settings.

In theory, organizational climate and capacity are particularly appropriate to examine in the context of health equity-oriented practice because of the importance of engaging in multifaceted, strategic approaches [14, 15], the need for a sustainable infrastructure for ongoing efforts [16], and the importance of working with partners internal and external to public health when intervening to impact the complex, systemic nature of health inequities [17]. However, the CFIR has not previously been specifically operationalized to measure implementation climate for equity-oriented practice. The purpose of the study was to develop a measure of equity-oriented implementation climate that may be used to gauge local public health systems’ capacity, identify areas for training, and track subsequent capacity improvements in order to evaluate the effectiveness of strategies to increase public health effectiveness in this area.

## Methods

### Data collection methods

A link to an online questionnaire (Qualtrics) was sent via email to all 115 LHD top administrators in Missouri. In addition to the original survey invitation, non-respondents received up two to three email reminders and a phone call. Responses were received from 92 participants for a final response rate of 80%). The survey was open from January 2016 to April 2016.

Approximately two weeks after the baseline questionnaire was completed, 52 selected administrators (based on willingness to participate as indicated in baseline survey) were sent a request to complete the questionnaire a second time as part of the test-retest study. Non-respondents received one email reminder. Responses were received from 28 participants for a response rate of 54%. The study protocol was approved by the Saint Louis University Institutional Review Board.

### Measures development

The questionnaire was developed based on theoretical frameworks and prior health equity initiatives in LHD settings using newly created and adaptations of existing items. The Consolidated Framework for Implementation Research (CFIR) provided an overarching framework for identifying relevant implementation domains both outside and within the LHD organizational setting [13]. One specific aim of the current study was to develop new items to measure implementation climate, described as a multi-domain construct and component of the inner organizational setting. Implementation climate is thought to represent the organization’s absorptive capacity and shared receptivity for change, and has been described as a tangible phenomenon (compared to less tangible “culture”) [13, 18]. Sub-domains of implementation climate include relative priority, tension for change, compatibility, organizational incentives and rewards, goals and feedback, and learning climate. Specifically, measurement items were worded to capture equity-oriented implementation climate by asking participants to rate each item in relation to practices and policies to address the needs of vulnerable populations.

The final questionnaire integrated implementation climate domains with previously developed measurement items characterizing aspects of LHD infrastructure and essential functions, adapted to the aims of the current project [19, 20]. We also examined the Bay Area Regional Health Inequities Initiative’s (BARHII) self-assessment tool domains, which focus on capacities needed to implement health equity initiatives (e.g, institutional commitment to address health inequities, understanding of social, environmental and structural determinants of health, structures that support true community partnerships, transparent & inclusive communication, hiring to address health inequities, community knowledge, collaboration skills, community organizing, and cultural competence/humility) [21]. Domains and constructs from these various sources were compared and contrasted, and a final set of items were selected to represent LHD organizational characteristics and capacities to address health equity and organized into three areas of practice: assessment and planning, monitoring and analysis, and demonstration of leadership support. A fourth practice area representing implementation of equity-oriented obesity prevention practices was included, which gauged the LHD’s role in obesity prevention through improving opportunities, policies and environments for health eating and physical activity among vulnerable populations. Obesity was chosen as a focus area based on its priority as a public health concern and its linkage to disparities across many leading causes of morbidity and mortality (e.g, heart disease, diabetes). Finally, areas for capacity building were captured by asking respondents to indicate specific items under each practice area where they would like to gain additional training.

Once the questionnaire was drafted, it was reviewed by three former LHD administrators to ensure construct validity of measurement items and to provide feedback on acceptability and feasibility of conducting the survey among the intended sample of current LHD administrators. Suggestions were provided for modifying wording to clarify individual questions, dropping questions to lessen the time burden involved with participating in the survey, and wording for the introductory email to request participation, with the questionnaire and data collection methods revised accordingly. The final questionnaire included measurement items representing four domains of implementation climate (16 items), and four practice areas: assessment and planning (9 items), monitoring and analysis (8 items), leadership support (11 items) and obesity prevention practice (7 items). The questionnaire is available in its entirety as a S1 Questionnaire.

### Analyses

Response scores from individual measurement items were summarized to create scales representing four separate domains of implementation climate, an overall implementation climate scale, and the four action areas (assessment and planning, monitoring and analysis, leadership support, and obesity prevention), with higher scores indicating more engagement. The resulting data were examined with descriptive statistics (frequency distributions for individual items and univariate statistics for summary scales). Linear regression models were used to examine whether higher implementation climate score was related to higher scores in the four action areas. Test-retest reliability was assessed by calculating intraclass correlation coefficients using the formula for 2-way, mixed effects [22]. Test-retest and descriptive analyses were conducted using IBM SPSS Statistics for Windows, Version 24.0. Regression analyses were conducted using SAS Software, Version 9.4.

## Results

When asked to identify the specific vulnerable populations in their jurisdictions, 100% of LHD respondents identified low income groups, followed in frequency by low education, elderly, and children/infants (Table 1). Participants were also asked to indicate areas where they would like to gain additional skills. The most frequently identified areas where additional skills and training were desired (representing between 70–76% of respondents) were incorporating social determinants frameworks and models, assessing the community needs and assets and health status of vulnerable populations in your community, incorporating evidence-based approaches to address the health of vulnerable populations, assessing access to care and health promoting resources among vulnerable populations, evaluating effectiveness of programs and changes in policies, systems, & environments, and evaluating the effectiveness of collaborations.

**Table 1:** LHD respondent identification of the vulnerable populations most in need of prevention and public health services in their jurisdiction.

Type of Vulnerable Population Identified	%
Poverty/low income	100
Low education	75.0
Elderly	75.0
Children/infants	71.7
Geographic area	64.1
Disability status	32.6
Racial/ethnic minorities	44.8
Immigrant populations	21.7
LGBT populations	9.8
Gender	5.4

Equity-oriented implementation climate was organized in four sub-domains: relative priority, tension for change, compatibility, and organizational incentives and rewards (Table 2). In terms of relative priority, most LHDs agreed that promoting the health of vulnerable populations was important and successfully prioritized; however, there was substantial disagreement over conflicts with other organizational priorities. With respect to tension for change, while most LHDs agreed there was a strong internal push to promote the health of vulnerable populations, respondents indicated gaps in a push for such efforts from the political arena and community. The strongest level of respondent agreement was observed for compatibility items, indicating programs and policies to promote the health of vulnerable populations are perceived to have a strong fit with organizational values and norms and existing practices. Likewise, respondents were in strong agreement that promoting the health of vulnerable populations was well regarded by leadership and aligned with other goals of the organization that could bring recognition (e.g., accreditation).

**Table 2:** LHD equity-oriented implementation climate: Distribution of item- and domain-specific responses (percent (%) and mean (Standard Deviation (S.D.)) and test-retest reliability (Intraclass Correlation Coefficients (ICC)).

	Strongly Agree/ Agree (%)	Mean (S.D.)	Item-specific ICC	Domain-specific* ICC
**Relative Priority**				0.55
Important relative to current initiatives	86.2	4.2 (0.66)	0.73	
Successfully prioritized	73.8	3.6 (0.71)	0.03	
Conflicts with other priorities	32.5	3.5 (0.88)	0.56	
**Tension for Change**				0.64
Internal push	66.7	3.9 (0.58)	0.59	
Political push	24.7	2.7 (0.92)	0.74	
Community push	39.5	3.1 (1.0)	0.64	
Funding requirement	61.7	4.1 (0.74)	0.44	
Essential to meet community need	88.9	3.4 (1.2)	0.64	
**Compatibility**				0.68
Organizational values/norms	91.4	4.3 (0.62)	0.63	
Other organizations values/norms	76.5	3.8 (0.70)	0.68	
Existing organizational practices	88.9	4.0 (0.55)	0.50	
Current programs	82.7	4.0 (0.75)	0.42	
**Organizational Incentives and Rewards**				0.85
Well regarded by the organization	85.2	4.0 (0.66)	0.72	
Teams/Individuals receive recognition	53.1	3.4 (0.78)	0.64	
Feedback influences goals	65.4	3.5 (0.59)	0.84	
Align with other organizational goals	70.4	3.7 (0.69)	0.76	

*ICC for overall implementation climate summary score = 0.82

Test-retest reliability results for the newly-developed implementation climate items indicated that most items had at least moderate to good reliability, with individual item ICC values ≥0.5 for thirteen and ≥0.7 for five of sixteen items. In addition, ICC for the four subdomains ranged from 0.55 to 0.85, and for the overall implementation climate score was 0.82.

Results from regression models examining the relationship between implementation climate and engagement in the four equity-oriented action areas are reported in Table 3. A higher index of implementation climate was related to a higher score in assessment and planning activities for vulnerable populations, and similarly related to other organizational characteristics such as analysis and monitoring, and leadership support. Implementation climate was also related to a higher score on the obesity prevention index. Relationships were not substantially altered and remained statistically significant after adjusting for population size of jurisdiction.

**Table 3:** Organizational implementation climate in relation to LHD equity-oriented action in four key practice areas: Assessment and planning, monitoring and analysis, leadership support, and obesity prevention (beta (p-value)).

	Equity-Oriented Assessment and Planning	Monitoring and Analysis of Data on Health Inequities	Leadership Support for Health Equity	Equity-Oriented Obesity Prevention
	Beta (p-value)	Beta (p-value)	Beta (p-value)	Beta (p-value)
Implementation climate, unadjusted	0.63 (0.0008)	0.28 (0.0014)	0.53 (0.0001)	1.60 (0.0001)
Implementation climate, adjusted for population size	0.69 (0.0006)	0.30 (0.0010)	0.54 (0.0001)	1.63 (0.0001)

## Discussion

Local public health departments are essential to the implementation of health equity initiatives, in large part, because they are poised to provide the needed contextualization in order to adapt practices to the vulnerable populations in their jurisdiction in collaboration with community members and other key partners across sectors. However, measurement tools are needed in order to assess capacity and identify specific organizational supports that bolster these health equity-oriented practices. The current study contributes to our understanding of structures and processes that may be identified as leverage points for implementation strategies designed to improve equity-oriented approaches in local public health departments [23].

Our findings further show that in LHDs where implementation climate is more supportive of health equity there is a higher likelihood of engagement in assessment, planning, and monitoring of activities specifically geared toward health equity in general and specifically with regard to obesity initiatives. LHDs in Missouri generally agreed with the importance of promoting the health of vulnerable populations and see a strong fit with organizational values and norms. However, there were frequently cited concerns about health equity initiatives conflicting with other organizational priorities, and the lack of external political and community support for this work. This may bode poorly for sustainability, if efforts to promote equity are seen as competing with other priorities rather than as being integrated with overall organizational strategy. Current national efforts, such as the national public health accreditation program, whose standards and measures include a number of items related to health equity may help to overcome some of this lack of local support and act as an external push toward more LHD equity-oriented implementation [9].

LHDs also highlighted the importance of a number of areas for future training. For example, cross-sector collaboration and recognition of gaps in capacity in this area was exemplified by the majority (74%) of respondents expressing the need to improve their ability to evaluate these collaborations. These findings point to the importance of training health department personnel so they have a better capacity for collaborative initiatives and enhanced understanding of health equity and social determinants as well as how they can translate these lessons to those outside the health arena in ways that enhance support rather than create opposition. The high frequency of respondents that indicated they wanted additional training and skills in the area of assessment and evaluation suggest this is another area for future training.

### Strengths and limitations

This is the first study that we are aware of that has developed a theory-based measure of implementation climate and examined it in relation to other areas of equity-oriented practice in local public health departments. Local health department organizational characteristics, including leadership engagement, and training needs, have been previously found to influence prioritization of strategies based in evidence, which may be more broadly construed to include equity-oriented practice [24]. Prior work has described the important role of the public health system in general and health departments in particular on intervening to reduce health inequities, which Liburd et al. describe as part of a three-way linkage between practice, policy and research [17]. The current study complements other studies that have examined the capacity of health departments to engage in health equity-related work that have been either qualitative in nature [25], have focused on other aspects of capacity [26–28], examined state rather than local settings [29], or require high levels of resources and commitment [21].

Our tool provides a way to take a snapshot in public health settings across a broad range of local communities. It does not, however provide a comprehensive organizational assessment, such as the tool created by the Bay Area Regional Health Inequities Initiative (BARHII) [21]. As such our tool might be considered more or a starting point for organizations that don’t have the capacity, readiness, or support for a more in-depth comprehensive review. Furthermore, as the measure is framed on domains of implementation climate it provides a way to gauge the organization’s receptivity to change with respect to equity-oriented practice, and thus may be used to guide targeted implementation strategies [30].

The study was conducted only in Missouri. In many ways, the findings could be more broadly applicable. The distribution of Missouri county LHD jurisdictions in the study sample is similar to that of US counties with respect to population size, with a slightly higher proportion of small-sized counties in the Missouri sample (57.6% of Missouri counties are <25,000, compared to 48.7% of all US counties). Within Missouri it was important to recognize that there are different vulnerable populations within LHD jurisdictions, and that this might influence LHD actions. It is noteworthy that the vulnerable populations that were identified most frequently by LHDs were low income, low education, the elderly and children. Future work might explore if this is due to LHDs responding with respect to objective assessment of community needs, or if other factors impact LHD capacity to recognize and address the needs across a broader set of vulnerable population groups. In addition, it is unclear if the tool might have different utility for LHDs that define the vulnerable populations in their jurisdiction that are most in need of prevention and public health services as being racial/ethnic minorities. Future studies might examine the utility of the tool for LHDs where racial equity might be the most appropriate lens. Further work could also aim to expand the size and geographic range of the sample and examine additional psychometric properties of the newly-developed measures in order to improve understanding and increase generalizability across contexts.

## Conclusion

In many ways public health as a field has valued the ability to understand the nature of relationships among variables over the ability to learn with communities how best to intervene to affect change [31] In order to improve local public health infrastructure and capacities to address health inequities it is important to first identify what these structures and capacities should be, develop ways to assess and track changes in these capacities, and garner practitioners’ perspectives on interventions for effective change. The results of this study suggest the newly developed tool may be considered for its utility across localities to gauge local public health organizational capacity for equity-oriented practice.

## Supporting information

S1 Appendix (DOCX)

S1 Data (XLSX)

S2 Data (XLSX)

S3 Data (DOCX)

S1 Questionnaire (DOCX)
