# Supplementary material for: Development of a measurement tool to assess local public health implementation climate and capacity for equity-oriented practice: Application to obesity prevention in a local public health system
Source: PLoS One. 2020 Sep 28;15(9):e0237380. doi: 10.1371/journal.pone.0237380 (PMC7521675; doi:10.1371/journal.pone.0237380)
Supplement: S3 Data — (DOCX) [file pone.0237380.s004.docx]

LHD Equity paper PLOS One recodes

**Data** recode;

set LHD equity climate PLOS One;

if Q14_3 = **1** then Q14_3_recode= **5**;

else if Q14_3 =**2** then Q14_3_recode= **4**;

else if Q14_3= **3** then Q14_3_recode= **3**;

else if Q14_3 =**4** then Q14_3_recode= **2**;

else if Q14_3 =**5** then Q14_3_recode= **1**;

RelativePriority= (Q14_1+ Q14_2+ Q14_3_recode);

Motivation= sum(of Q15_1 Q15_2 Q15_3 Q15_4 Q15_5);

ProgramFit= Sum(of Q16_1 Q16_2 Q16_3 Q16_4);

OrgSupport= Sum(of Q17_1 Q17_2 Q17_4 Q17_5);

ImpClim= Sum(of RelativePriority Motivation ProgramFit OrgSupport);

If impclim <= **53** then impclim_cat =**1**;

if (impclim <=**60**) and (impclim>**53**) then impclim_cat=**2**;

if (impclim <=**66**) and (impclim>**60**) then impclim_cat=**3**;

if impclim > **66** then impclim_cat=**4**;

If impclim = missing then impclim_cat= '';

/*Recode Assessment/Planning Variables*/

if Q6_1_1 <=**2** then Q6_1_1_Cat =**1**;

else if Q6_1_1 = **3** then Q6_1_1_Cat =**2**;

Else if Q6_1_1 >=**4** then Q6_1_1_Cat=**3**;

else if Q6_1_1 ='.' then Q6_1_1_Cat=**3**;

if Q6_1_2 <=**2** then Q6_1_2_Cat =**1**;

else if Q6_1_2 = **3** then Q6_1_2_Cat =**2**;

Else if Q6_1_2 >=**4** then Q6_1_2_Cat=**3**;

else if Q6_1_2 = '.' then Q6_1_2_Cat=**3**;

if Q6_1_3 <=**2** then Q6_1_3_Cat =**1**;

else if Q6_1_3 = **3** then Q6_1_3_Cat =**2**;

Else if Q6_1_3 >=**4** then Q6_1_3_Cat=**3**;

else if Q6_1_3 = '.' then Q6_1_3_Cat=**3**;

if Q6_1_4 <=**2** then Q6_1_4_Cat =**1**;

else if Q6_1_4 = **3** then Q6_1_4_Cat =**2**;

Else if Q6_1_4 >=**4** then Q6_1_4_Cat=**3**;

else if Q6_1_4 = '.' then Q6_1_4_Cat=**3**;

if Q6_1_5 <=**2** then Q6_1_5_Cat =**1**;

else if Q6_1_5 = **3** then Q6_1_5_Cat =**2**;

Else if Q6_1_5 >=**4** then Q6_1_5_Cat=**3**;

else if Q6_1_5 = '.' then Q6_1_5_Cat=**3**;

if Q6_1_6 <=**2** then Q6_1_1_Cat =**1**;

else if Q6_1_6 = **3** then Q6_1_6_Cat =**2**;

Else if Q6_1_6 >=**4** then Q6_1_6_Cat=**3**;

else if Q6_1_6 = '.' then Q6_1_6_Cat=**3**;

if Q6_1_7 <=**2** then Q6_1_7_Cat =**1**;

else if Q6_1_7 = **3** then Q6_1_7_Cat =**2**;

Else if Q6_1_7 >=**4** then Q6_1_7_Cat=**3**;

else if Q6_1_7 = '.' then Q6_1_7_Cat=**3**;

if Q6_1_8 <=**2** then Q6_1_8_Cat =**1**;

else if Q6_1_8 = **3** then Q6_1_8_Cat =**2**;

Else if Q6_1_8 >=**4** then Q6_1_8_Cat=**3**;

else if Q6_1_8 = '.' then Q6_1_8_Cat=**3**;

if Q6_1_9 <=**2** then Q6_1_9_Cat =**1**;

else if Q6_1_9 = **3** then Q6_1_9_Cat =**2**;

Else if Q6_1_9 >=**4** then Q6_1_9_Cat=**3**;

else if Q6_1_9 = '.' then Q6_1_9_Cat= **3**;

*/ Composite Assessment variable*/;

Assessment= Sum(of Q6_1_1 Q6_1_2 Q6_1_3 Q6_1_4 Q6_1_5 Q6_1_6 Q6_1_7 Q6_1_8 Q6_1_9);

/*Recode Monitor/Analyze Variables*/

if Q10_1_1 =**1** then Q10_1_1_Cat=**1**;

else if Q10_1_1 =**2** or Q10_1_1 =**3** then Q10_1_1_Cat= **2**;

else if Q10_1_1 >=**4** then Q10_1_1_Cat = **3**;

else if Q10_1_1 ='.' then Q10_1_1_Cat=**3**;

if Q10_1_2 =**1** then Q10_1_2_Cat=**1**;

else if Q10_1_2 =**2** or Q10_1_2 =**3** then Q10_1_2_Cat= **2**;

else if Q10_1_2 >=**4** then Q10_1_2_Cat = **3**;

else if Q10_1_2 ='.' then Q10_1_2_Cat=**3**;

if Q10_1_3 =**1** then Q10_1_3_Cat=**1**;

else if Q10_1_3 =**2** or Q10_1_3 =**3** then Q10_1_3_Cat= **2**;

else if Q10_1_3 >=**4** then Q10_1_3_Cat = **3**;

else if Q10_1_3 ='.' then Q10_1_3_Cat=**3**;

if Q10_1_4 =**1** then Q10_1_4_Cat=**1**;

else if Q10_1_4 =**2** or Q10_1_4 =**3** then Q10_1_4_Cat= **2**;

else if Q10_1_4 >=**4** then Q10_1_4_Cat = **3**;

else if Q10_1_4 ='.' then Q10_1_4_Cat=**3**;

if Q10_1_5 =**1** then Q10_1_5_Cat=**1**;

else if Q10_1_5 =**2** or Q10_1_5 =**3** then Q10_1_5_Cat= **2**;

else if Q10_1_5 >=**4** then Q10_1_5_Cat = **3**;

else if Q10_1_5 ='.' then Q10_1_5_Cat=**3**;

if Q10_1_6 =**1** then Q10_1_6_Cat=**1**;

else if Q10_1_6 =**2** or Q10_1_6 =**3** then Q10_1_6_Cat= **2**;

else if Q10_1_6 >=**4** then Q10_1_6_Cat = **3**;

else if Q10_1_6 ='.' then Q10_1_6_Cat=**3**;

if Q10_1_7 =**1** then Q10_1_7_Cat=**1**;

else if Q10_1_7 =**2** or Q10_1_7 =**3** then Q10_1_7_Cat= **2**;

else if Q10_1_7 >=**4** then Q10_1_7_Cat = **3**;

else if Q10_1_7 ='.' then Q10_1_7_Cat=**3**;

if Q10_1_8 =**1** then Q10_1_8_Cat=**1**;

else if Q10_1_8 =**2** or Q10_1_8 =**3** then Q10_1_8_Cat= **2**;

else if Q10_1_8 >=**4** then Q10_1_8_Cat = **3**;

else if Q10_1_8 ='.' then Q10_1_8_Cat=**3**;

if Q10_1_9 =**1** then Q10_1_9_Cat=**1**;

else if Q10_1_9 =**2** or Q10_1_9 =**3** then Q10_1_9_Cat= **2**;

else if Q10_1_9 >=**4** then Q10_1_9_Cat = **3**;

else if Q10_1_9 ='.' then Q10_1_9_Cat=**3**;

*/Create Composite Montitor Score*/;

Monitor= Sum (of Q10_1_1 Q10_1_2 Q10_1_3 Q10_1_4

Q10_1_5 Q10_1_6 Q10_1_7 Q10_1_8 Q10_1_9);

/* Recode Variables in Leadership*/

if Q11_1_1 =**1** then Q11_1_1_Cat=**1**;

else if Q11_1_1 =**2** or Q11_1_1 =**3** then Q11_1_1_Cat= **2**;

else if Q11_1_1 >=**4** then Q11_1_1_Cat = **3**;

else if Q11_1_1 ='.' then Q11_1_1_Cat=**3**;

if Q11_1_2 =**1** then Q11_1_2_Cat=**1**;

else if Q11_1_2 =**2** or Q11_1_2 =**3** then Q11_1_2_Cat= **2**;

else if Q11_1_2 >=**4** then Q11_1_2_Cat = **3**;

else if Q11_1_2 ='.' then Q11_1_2_Cat=**3**;

if Q11_1_3 =**1** then Q11_1_3_Cat=**1**;

else if Q11_1_3 =**2** or Q11_1_3 =**3** then Q11_1_3_Cat= **2**;

else if Q11_1_3 >=**4** then Q11_1_3_Cat = **3**;

else if Q11_1_3 ='.' then Q11_1_3_Cat=**3**;

if Q11_1_4 =**1** then Q11_1_4_Cat=**1**;

else if Q11_1_4 =**2** or Q11_1_4 =**3** then Q11_1_4_Cat= **2**;

else if Q11_1_4 >=**4** then Q11_1_4_Cat = **3**;

else if Q11_1_4 ='.' then Q11_1_4_Cat=**3**;

if Q11_1_5 =**1** then Q11_1_5_Cat=**1**;

else if Q11_1_5 =**2** or Q11_1_5 =**3** then Q11_1_5_Cat= **2**;

else if Q11_1_5 >=**4** then Q11_1_5_Cat = **3**;

else if Q11_1_5 ='.' then Q11_1_5_Cat=**3**;

if Q11_1_6 =**1** then Q11_1_6_Cat=**1**;

else if Q11_1_6 =**2** or Q11_1_6 =**3** then Q11_1_6_Cat= **2**;

else if Q11_1_6 >=**4** then Q11_1_6_Cat = **3**;

else if Q11_1_6 ='.' then Q11_1_6_Cat=**3**;

if Q11_1_7 =**1** then Q11_1_7_Cat=**1**;

else if Q11_1_7 =**2** or Q11_1_7 =**3** then Q11_1_7_Cat= **2**;

else if Q11_1_7 >=**4** then Q11_1_7_Cat = **3**;

else if Q11_1_7 ='.' then Q11_1_7_Cat=**3**;

if Q11_1_8 =**1** then Q11_1_8_Cat=**1**;

else if Q11_1_8 =**2** or Q11_1_8 =**3** then Q11_1_8_Cat= **2**;

else if Q11_1_8 >=**4** then Q11_1_8_Cat = **3**;

else if Q11_1_8 ='.' then Q11_1_8_Cat=**3**;

if Q11_1_9 =**1** then Q11_1_9_Cat=**1**;

else if Q11_1_9 =**2** or Q11_1_9 =**3** then Q11_1_9_Cat= **2**;

else if Q11_1_9 >=**4** then Q11_1_9_Cat = **3**;

else if Q11_1_9 ='.' then Q11_1_9_Cat=**3**;

if Q11_1_10 =**1** then Q11_1_10_Cat=**1**;

else if Q11_1_10 =**2** or Q11_1_10 =**3** then Q11_1_10_Cat= **2**;

else if Q11_1_10 >=**4** then Q11_1_10_Cat = **3**;

else if Q11_1_10 ='.' then Q11_1_10_Cat=**3**;

if Q11_1_11 =**1** then Q11_1_11_Cat=**1**;

else if Q11_1_11 =**2** or Q11_1_11 =**3** then Q11_1_11_Cat= **2**;

else if Q11_1_11 >=**4** then Q11_1_11_Cat = **3**;

else if Q11_1_11 ='.' then Q11_1_11_Cat=**3**;

*/ Recode Q20_1 - Q20_7 as any role and no role*/;

If (Q20_1_1= **1**) or (Q20_1_2= **1**) then Q20_1_cat=**1**;

else if Q20_1_4= **1** then Q20_1_cat=**1**;

else if Q20_1_3=**1** then Q20_1_cat=**0**;

If (Q20_2_1= **1**) or (Q20_2_2= **1**) then Q20_2_cat=**1**;

else if Q20_2_4= **1** then Q20_2_cat=**1**;

else if Q20_2_3=**1** then Q20_2_cat=**0**;

If (Q20_3_1= **1**) or (Q20_3_2= **1**) then Q20_3_cat=**1**;

else if Q20_3_4= **1** then Q20_3_cat=**1**;

else if Q20_3_3=**1** then Q20_3_cat=**0**;

If (Q20_4_1= **1**) or (Q20_4_2= **1**) then Q20_4_cat=**1**;

else if Q20_4_4= **1** then Q20_4_cat=**1**;

else if Q20_4_3=**1** then Q20_4_cat=**0**;

If (Q20_5_1= **1**) or (Q20_5_2= **1**) then Q20_5_cat=**1**;

else if Q20_5_4= **1** then Q20_5_cat=**1**;

else if Q20_5_3=**1** then Q20_5_cat=**0**;

If (Q20_6_1= **1**) or (Q20_6_2= **1**) then Q20_6_cat=**1**;

else if Q20_6_4= **1** then Q20_6_cat=**1**;

else if Q20_6_3=**1** then Q20_6_cat=**0**;

If (Q20_7_1= **1**) or (Q20_7_2= **1**) then Q20_7_cat=**1**;

else if Q20_7_4= **1** then Q20_7_cat=**1**;

else if Q20_7_3=**1** then Q20_7_cat=**0**;

*/ Q20 variables Recode with Don't Know as no role*/;

If (Q20_1_1= **1**) or (Q20_1_2= **1**) then Q20_1_cat2=**1**;

else if Q20_1_4= **1** then Q20_1_cat2=**0**;

else if Q20_1_3=**1** then Q20_1_cat2=**0**;

If (Q20_2_1= **1**) or (Q20_2_2= **1**) then Q20_2_cat2=**1**;

else if Q20_2_4= **1** then Q20_2_cat2=**0**;

else if Q20_2_3=**1** then Q20_2_cat2=**0**;

If (Q20_3_1= **1**) or (Q20_3_2= **1**) then Q20_3_cat2=**1**;

else if Q20_3_4= **1** then Q20_3_cat2=**0**;

else if Q20_3_3=**1** then Q20_3_cat2=**0**;

If (Q20_4_1= **1**) or (Q20_4_2= **1**) then Q20_4_cat2=**1**;

else if Q20_4_4= **1** then Q20_4_cat2=**0**;

else if Q20_4_3=**1** then Q20_4_cat2=**0**;

If (Q20_5_1= **1**) or (Q20_5_2= **1**) then Q20_5_cat2=**1**;

else if Q20_5_4= **1** then Q20_5_cat2=**0**;

else if Q20_5_3=**1** then Q20_5_cat2=**0**;

If (Q20_6_1= **1**) or (Q20_6_2= **1**) then Q20_6_cat2=**1**;

else if Q20_6_4= **1** then Q20_6_cat2=**0**;

else if Q20_6_3=**1** then Q20_6_cat2=**0**;

If (Q20_7_1= **1**) or (Q20_7_2= **1**) then Q20_7_cat2=**1**;

else if Q20_7_4= **1** then Q20_7_cat2=**0**;

else if Q20_7_3=**1** then Q20_7_cat2=**0**;

*/Create a summary variable of obesity prevention leadership*/;

Q20_Summary= sum(of Q20_1_cat2 Q20_2_cat2 Q20_3_cat2 Q20_4_cat2 Q20_5_cat2 Q20_6_cat2 Q20_7_cat2);

*/Reverse coded Q6 Variables and combined Don't Know and Never, and within the past

year and 1-3 years, lead in question Q5 was accounted for*/;

If (Q6_1_1=**4**) or (Q6_1_1=**5**) then Q6_1_1Cat= **0**;

Else if Q6_1_1=**3** then Q6_1_1Cat= **1**;

Else if (Q6_1_1=**2**) or (Q6_1_1=**1**) then Q6_1_1Cat=**2**;

else if Q5= **2** then Q6_1_1cat=**0**;

If (Q6_1_2=**4**) or (Q6_1_2=**5**) then Q6_1_2Cat= **0**;

Else if Q6_1_2=**3** then Q6_1_2Cat= **1**;

Else if (Q6_1_2=**2**) or (Q6_1_2=**1**) then Q6_1_2Cat=**2**;

else if Q5= **2** then Q6_1_2cat=**0**;

If (Q6_1_3=**4**) or (Q6_1_3=**5**) then Q6_1_3Cat= **0**;

Else if Q6_1_3=**3** then Q6_1_3Cat= **1**;

Else if (Q6_1_3=**2**) or (Q6_1_3=**1**) then Q6_1_3Cat=**2**;

else if Q5= **2** then Q6_1_3cat=**0**;

If (Q6_1_4=**4**) or (Q6_1_4=**5**) then Q6_1_4Cat= **0**;

Else if Q6_1_4=**3** then Q6_1_4Cat= **1**;

Else if (Q6_1_4=**2**) or (Q6_1_4=**1**) then Q6_1_4Cat=**2**;

else if Q5= **2** then Q6_1_4cat=**0**;

If (Q6_1_5=**4**) or (Q6_1_5=**5**) then Q6_1_5Cat= **0**;

Else if Q6_1_5=**3** then Q6_1_5Cat= **1**;

Else if (Q6_1_5=**2**) or (Q6_1_5=**1**) then Q6_1_5Cat=**2**;

else if Q5= **2** then Q6_1_5cat=**0**;

If (Q6_1_6=**4**) or (Q6_1_6=**5**) then Q6_1_6Cat= **0**;

Else if Q6_1_6=**3** then Q6_1_6Cat= **1**;

Else if (Q6_1_6=**2**) or (Q6_1_6=**1**) then Q6_1_6Cat=**2**;

else if Q5= **2** then Q6_1_6cat=**0**;

If (Q6_1_7=**4**) or (Q6_1_7=**5**) then Q6_1_7Cat= **0**;

Else if Q6_1_7=**3** then Q6_1_7Cat= **1**;

Else if (Q6_1_7=**2**) or (Q6_1_7=**1**) then Q6_1_7Cat=**2**;

else if Q5= **2** then Q6_1_7cat=**0**;

If (Q6_1_8=**4**) or (Q6_1_8=**5**) then Q6_1_8Cat= **0**;

Else if Q6_1_8=**3** then Q6_1_8Cat= **1**;

Else if (Q6_1_8=**2**) or (Q6_1_8=**1**) then Q6_1_8Cat=**2**;

else if Q5= **2** then Q6_1_8cat=**0**;

If (Q6_1_9=**4**) or (Q6_1_9=**5**) then Q6_1_9Cat= **0**;

Else if Q6_1_9=**3** then Q6_1_9Cat= **1**;

Else if (Q6_1_9=**2**) or (Q6_1_9=**1**) then Q6_1_9Cat=**2**;

else if Q5= **2** then Q6_1_9cat=**0**;

*/ Q10 variables were recoded with Don't know and never combined and the coding was reversed

The lead in question Q9 was accounted for*/;

If (Q10_1_1=**5**) or (Q10_1_1=**4**) then Q10_1_1Cat=**0**;

else If Q10_1_1= **3** then Q10_1_1Cat=**1**;

else If Q10_1_1=**2** then Q10_1_1Cat=**2**;

else If Q10_1_1=**1** then Q10_1_1Cat=**3**;

else If Q9=**2** then Q10_1_1Cat=**0**;

If (Q10_1_2=**5**) or (Q10_1_2=**4**) then Q10_1_2Cat=**0**;

else If Q10_1_2= **3** then Q10_1_2Cat=**1**;

else If Q10_1_2=**2** then Q10_1_2Cat=**2**;

else If Q10_1_2=**1** then Q10_1_2Cat=**3**;

else If Q9=**2** then Q10_1_2Cat=**0**;

If (Q10_1_3=**5**) or (Q10_1_3=**4**) then Q10_1_3Cat=**0**;

else If Q10_1_3= **3** then Q10_1_3Cat=**1**;

else If Q10_1_3=**2** then Q10_1_3Cat=**2**;

else If Q10_1_3=**1** then Q10_1_3Cat=**3**;

else If Q9=**2** then Q10_1_3Cat=**0**;

If (Q10_1_4=**5**) or (Q10_1_4=**4**) then Q10_1_4Cat=**0**;

else If Q10_1_4= **3** then Q10_1_4Cat=**1**;

else If Q10_1_4=**2** then Q10_1_4Cat=**2**;

else If Q10_1_4=**1** then Q10_1_4Cat=**3**;

else If Q9=**2** then Q10_1_4Cat=**0**;

If (Q10_1_5=**5**) or (Q10_1_5=**4**) then Q10_1_5Cat=**0**;

else If Q10_1_5= **3** then Q10_1_5Cat=**1**;

else If Q10_1_5=**2** then Q10_1_5Cat=**2**;

else If Q10_1_5=**1** then Q10_1_5Cat=**3**;

else If Q9=**2** then Q10_1_5Cat=**0**;

If (Q10_1_6=**5**) or (Q10_1_6=**4**) then Q10_1_6Cat=**0**;

else If Q10_1_6= **3** then Q10_1_6Cat=**1**;

else If Q10_1_6=**2** then Q10_1_6Cat=**2**;

else If Q10_1_6=**1** then Q10_1_6Cat=**3**;

else If Q9=**2** then Q10_1_6Cat=**0**;

If (Q10_1_7=**5**) or (Q10_1_7=**4**) then Q10_1_7Cat=**0**;

else If Q10_1_7= **3** then Q10_1_7Cat=**1**;

else If Q10_1_7=**2** then Q10_1_7Cat=**2**;

else If Q10_1_7=**1** then Q10_1_7Cat=**3**;

else If Q9=**2** then Q10_1_7Cat=**0**;

If (Q10_1_8=**5**) or (Q10_1_8=**4**) then Q10_1_8Cat=**0**;

else If Q10_1_8= **3** then Q10_1_8Cat=**1**;

else If Q10_1_8=**2** then Q10_1_8Cat=**2**;

else If Q10_1_8=**1** then Q10_1_8Cat=**3**;

else If Q9=**2** then Q10_1_8Cat=**0**;

*/Reverse coded Q11 and combined Don't Know and Never*/;

If (Q11_1_1=**5**) or (Q11_1_1=**4**) then Q11_1_1Cat=**0**;

else If Q11_1_1= **3** then Q11_1_1Cat=**1**;

else If Q11_1_1=**2** then Q11_1_1Cat=**2**;

else If Q11_1_1=**1** then Q11_1_1Cat=**3**;

If (Q11_1_2=**5**) or (Q11_1_2=**4**) then Q11_1_2Cat=**0**;

else If Q11_1_2= **3** then Q11_1_2Cat=**1**;

else If Q11_1_2=**2** then Q11_1_2Cat=**2**;

else If Q11_1_2=**1** then Q11_1_2Cat=**3**;

If (Q11_1_3=**5**) or (Q11_1_3=**4**) then Q11_1_3Cat=**0**;

else If Q11_1_3= **3** then Q11_1_3Cat=**1**;

else If Q11_1_3=**2** then Q11_1_3Cat=**2**;

else If Q11_1_3=**1** then Q11_1_3Cat=**3**;

If (Q11_1_4=**5**) or (Q11_1_4=**4**) then Q11_1_4Cat=**0**;

else If Q11_1_4= **3** then Q11_1_4Cat=**1**;

else If Q11_1_4=**2** then Q11_1_4Cat=**2**;

else If Q11_1_4=**1** then Q11_1_4Cat=**3**;

If (Q11_1_5=**5**) or (Q11_1_5=**4**) then Q11_1_5Cat=**0**;

else If Q11_1_5= **3** then Q11_1_5Cat=**1**;

else If Q11_1_5=**2** then Q11_1_5Cat=**2**;

else If Q11_1_5=**1** then Q11_1_5Cat=**3**;

If (Q11_1_6=**5**) or (Q11_1_6=**4**) then Q11_1_6Cat=**0**;

else If Q11_1_6= **3** then Q11_1_6Cat=**1**;

else If Q11_1_6=**2** then Q11_1_6Cat=**2**;

else If Q11_1_6=**1** then Q11_1_6Cat=**3**;

If (Q11_1_7=**5**) or (Q11_1_7=**4**) then Q11_1_7Cat=**0**;

else If Q11_1_7= **3** then Q11_1_7Cat=**1**;

else If Q11_1_7=**2** then Q11_1_7Cat=**2**;

else If Q11_1_7=**1** then Q11_1_7Cat=**3**;

If (Q11_1_8=**5**) or (Q11_1_8=**4**) then Q11_1_8Cat=**0**;

else If Q11_1_8= **3** then Q11_1_8Cat=**1**;

else If Q11_1_8=**2** then Q11_1_8Cat=**2**;

else If Q11_1_8=**1** then Q11_1_8Cat=**3**;

If (Q11_1_9=**5**) or (Q11_1_9=**4**) then Q11_1_9Cat=**0**;

else If Q11_1_9= **3** then Q11_1_9Cat=**1**;

else If Q11_1_9=**2** then Q11_1_9Cat=**2**;

else If Q11_1_9=**1** then Q11_1_9Cat=**3**;

If (Q11_1_10=**5**) or (Q11_1_10=**4**) then Q11_1_10Cat=**0**;

else If Q11_1_10= **3** then Q11_1_10Cat=**1**;

else If Q11_1_10=**2** then Q11_1_10Cat=**2**;

else If Q11_1_10=**1** then Q11_1_10Cat=**3**;

If (Q11_1_11=**5**) or (Q11_1_11=**4**) then Q11_1_11Cat=**0**;

else If Q11_1_11= **3** then Q11_1_11Cat=**1**;

else If Q11_1_11=**2** then Q11_1_11Cat=**2**;

else If Q11_1_11=**1** then Q11_1_11Cat=**3**;

*/Create a summary variable for the Q6 Variables Using the recoded Q6Cat*/;

Q6_Summary= Sum (of Q6_1_1Cat Q6_1_2Cat Q6_1_3Cat Q6_1_4Cat Q6_1_5Cat

Q6_1_6Cat Q6_1_7Cat Q6_1_8cat);

*/Create a summary variable for the Q10 Variables Using the Recoded Q10Cat variables*/;

Q10_Summary= Sum (of Q10_1_1Cat Q10_1_2Cat Q10_1_3Cat Q10_1_4Cat Q10_1_5Cat

Q10_1_6Cat Q10_1_7Cat Q10_1_8Cat);

*/Create a summary variable for the Q11 Variables Using Recoded Q11Cat Variables*/;

Q11_Summary= Sum (of Q11_1_1Cat Q11_1_2Cat Q11_1_3Cat Q11_1_4Cat Q11_1_5Cat Q11_1_6Cat

Q11_1_7Cat Q11_1_8Cat Q11_1_9Cat Q11_1_10Cat Q11_1_11Cat);

**run**;
