# Supplementary material for: Development of a measurement tool to assess local public health implementation climate and capacity for equity-oriented practice: Application to obesity prevention in a local public health system
Source: PLoS One. 2020 Sep 28;15(9):e0237380. doi: 10.1371/journal.pone.0237380 (PMC7521675; doi:10.1371/journal.pone.0237380)
Supplement: S1 Questionnaire — (DOCX) [file pone.0237380.s005.docx]

**Survey on Local Health Department Efforts to Reduce Disparities and Enhance the Health of Vulnerable Populations**

The purpose of this survey is to gain some understanding of local public health agency (LPHA) efforts to promote the health of vulnerable populations in Missouri. Specifically, we would like to know which vulnerable populations you work with, the kinds of policies and programs you have put into place to reduce disparities and enhance health among these populations, and what kinds of skills and resources would be helpful to support work in this area. We hope that the findings from this survey will help us develop training opportunities for public health professionals in Missouri and elsewhere. Please note that it is very useful to get responses from all Missouri LPHAs in order to represent a broad mix of activities and approaches across Missouri communities.

There are 4 sections in the survey, and based on information from pilot testers, should take 10-15 minutes to complete. It may take you more or less time depending on your responses. Please note that all data will be kept confidential and published or shared data will ONLY be reported in aggregate numbers. As a thank you for taking part in the survey, we are offering you a $20 gift card at the end of the survey.

There are many other terms that may be applied to refer to vulnerable populations, and we recognize that the types of vulnerable populations and priority health issues will vary by community. The Centers for Disease Control and Prevention (CDC) uses the term “vulnerable populations” to describe groups who are at risk of health disparities, including groups defined by race/ethnicity, socio-economic status, geography, gender, age, disability status, and LGBT identity.

| **Q2_1** | Racial/ethnic minorities | **Checked = 1**  **Unchecked = “.”** |
| --- | --- | --- |
| **Q2_2** | Poverty/low income | **Checked = 1**  **Unchecked = “.”** |
| **Q2_3** | Low education | **Checked = 1**  **Unchecked = “.”** |
| **Q2_4** | Geographic area (e.g., remote areas, low-resource neighborhoods) | **Checked = 1**  **Unchecked = “.”** |
| **Q2_5** | Elderly | **Checked = 1**  **Unchecked = “.”** |
| **Q2_6** | Children/infants | **Checked = 1**  **Unchecked = “.”** |
| **Q2_7** | Disability status | **Checked = 1**  **Unchecked = “.”** |
| **Q2_8** | Gender | **Checked = 1**  **Unchecked = “.”** |
| **Q2_9** | LGBT populations | **Checked = 1**  **Unchecked = “.”** |
| **Q2_10** | Immigrant populations | **Checked = 1**  **Unchecked = “.”** |
| **Q2_11** | Other | **Checked = 1**  **Unchecked = “.”** |
| **Q2_11_TEXT** | Other | **None** |

1. Please indicate the vulnerable populations most in need of prevention and public health services in your jurisdiction. Check all that apply.

**Q3.** For the remainder of this survey, please keep in mind the vulnerable populations you identified in question 1 as you answer the following questions.

**Part I.** Characteristics of LPHA

1. **Q4.** Does your LPHA have a formal, written strategy in your strategic plan and/or action plan that specifically addresses vulnerable populations?

| Yes | **1** |
| --- | --- |
| No | **2** |
| Don’t Know | **3** |

1. **Q5.** Does your LPHA include consideration of vulnerable populations when conducting community health assessment and/or community health improvement planning?

| Yes | **1** |
| --- | --- |
| No | **2** |
| Don’t Know | **3** |

**If YES or DK,**

2a. Please tell us how frequently your LPHA engages in any of the following assessment and planning activities related to the health of vulnerable populations in your community and if you would like to gain skills or improve in this area.

Please choose within the past year, 1-3 years ago, 3 or more years ago, never or don’t know, and then indicate if you/your staff would like to gain skills or improve in this area.

|  |  |  | **Gain Skills?** |
| --- | --- | --- | --- |
| **Q6_1_1** | Assess the health status of vulnerable populations in your community | **1 = Within the past year**  **2 = 1-3 years ago**  **3 = 3 or more years ago**  **4 = Never**  **5 = Don't Know** | **Q6_2_1:**  **1 = Yes**  **2 = No** |
| **Q6_1_2** | Identify the extent to which vulnerable populations have reduced access to medical and/or other health-promoting resources | **1 = Within the past year**  **2 = 1-3 years ago**  **3 = 3 or more years ago**  **4 = Never**  **5 = Don't Know** | **Q6_2_2:**  **1 = Yes**  **2 = No** |
| **Q6_1_3** | Involve community partners and other local agencies/sectors that represent vulnerable populations | **1 = Within the past year**  **2 = 1-3 years ago**  **3 = 3 or more years ago**  **4 = Never**  **5 = Don't Know** | **Q6_2_3:**  **1 = Yes**  **2 = No** |
| **Q6_1_4** | Engage local policymakers and other stakeholders in plans to address the health of vulnerable populations | **1 = Within the past year**  **2 = 1-3 years ago**  **3 = 3 or more years ago**  **4 = Never**  **5 = Don't Know** | **Q6_2_4:**  **1 = Yes**  **2 = No** |
| **Q6_1_5** | Incorporate social determinants^[[1]](#footnote-1)^ frameworks and models | **1 = Within the past year**  **2 = 1-3 years ago**  **3 = 3 or more years ago**  **4 = Never**  **5 = Don't Know** | **Q6_2_5:**  **1 = Yes**  **2 = No** |
| **Q6_1_6** | Assess community needs and assets for addressing the health of vulnerable populations~~,~~ in terms of resources, policy environment, and/or public perceptions | **1 = Within the past year**  **2 = 1-3 years ago**  **3 = 3 or more years ago**  **4 = Never**  **5 = Don't Know** | **Q6_2_6:**  **1 = Yes**  **2 = No** |
| **Q6_1_7** | Prioritize, identify or develop laws, policies and ordinances to address the health of vulnerable populations | **1 = Within the past year**  **2 = 1-3 years ago**  **3 = 3 or more years ago**  **4 = Never**  **5 = Don't Know** | **Q6_2_7:**  **1 = Yes**  **2 = No** |
| **Q6_1_8** | Incorporate evidence-based approaches^[[2]](#footnote-2)^ to address the health of vulnerable populations | **1 = Within the past year**  **2 = 1-3 years ago**  **3 = 3 or more years ago**  **4 = Never**  **5 = Don't Know** | **Q6_2_8:**  **1 = Yes**  **2 = No** |
| **Q6_1_9** | Identify potential funding sources for supporting interventions related to the health of vulnerable populations | **1 = Within the past year**  **2 = 1-3 years ago**  **3 = 3 or more years ago**  **4 = Never**  **5 = Don't Know** | **Q6_2_9:**  **1 = Yes**  **2 = No** |
| **Q6_1_10** | Other (please specify) | **1 = Yes**  **2 = No** | **Q6_2_10:**  **1 = Yes**  **2 = No** |
| **Q6_1_10_TEXT** | Other (please specify) - TEXT | **None** | **Q6_2_10_TEXT:**  **None** |

1. **Q7.** Does your LPHA have programs and policies that promote the health of vulnerable populations:

| In all programs and policies | **1** |
| --- | --- |
| In some programs and policies | **2** |
| In no programs and policies | **3** |

3a. If “ALL” or “SOME”]: Please list the top 1-3 programs and/or policy areas that have

been designed to promote the health of vulnerable populations:

_____________________________________________________________

_____________________________________________________________

_____________________________________________________________

| **Q8_1_TEXT** | **None** |
| --- | --- |
| **ProgArea1** | **1 = WIC/Maternal Child Health**  **2 = Immunizations**  **3 = Health education/healthy eating/active living/health promotion**  **4 = Home health**  **5 = Free/reduced cost clinics/screenings**  **6 = Emergency preparedness**  **7 = Smoking prevention/cessation**  **8 = Chronic disease management**  **9 = Other** |
| **Q8_2_TEXT** | **None** |
| **ProgArea2** | **1 = WIC/Maternal Child Health**  **2 = Immunizations**  **3 = Health education/healthy eating/active living/health promotion**  **4 = Home health**  **5 = Free/reduced cost clinics/screenings**  **6 = Emergency preparedness**  **7 = Smoking prevention/cessation**  **8 = Chronic disease management**  **9 = Other** |
| **Q8_3_TEXT** | **None** |
| **ProgArea3** | **1 = WIC/Maternal Child Health**  **2 = Immunizations**  **3 = Health education/healthy eating/active living/health promotion**  **4 = Home health**  **5 = Free/reduced cost clinics/screenings**  **6 = Emergency preparedness**  **7 = Smoking prevention/cessation**  **8 = Chronic disease management**  **9 = Other** |

**Q9.** Does your LPHA monitor the health and/or analyze data related to the health of vulnerable

populations?

Please note that while this question may seem similar to previous questions, here we are asking

about activities that may occur in a more ongoing manner than community health assessment

and/or improvement planning activities.

| Yes | **1** |
| --- | --- |
| No | **2** |
| Don’t Know | **3** |

**If YES or DK,**

4a. Please tell us in which of the following ways your LPHA monitors and analyzes the health of vulnerable populations. Again, please note that while these questions may seem similar to previous questions, here we are asking about activities that may occur in a more ongoing manner than community health assessment and/or improvement planning activities.

Please choose either always/often, sometimes, rarely or never in response to each item, and then

indicate if you/your staff would like to gain skills or improve in this area by checking all that apply.

|  |  | **Checked = 1**  **Unchecked = “.”** | **Gain Skills?** |
| --- | --- | --- | --- |
| **Q10_1_1** | Obtain data necessary for assessing changes in the health of vulnerable populations | **1 = Always/often**  **2 = Sometimes**  **3 = Rarely**  **4 = Never**  **5 = Don't Know** | **Q10_2_1:**  **1 = Yes**  **2 = No** |
| **Q10_1_2** | Conduct analyses of local level data^[[3]](#footnote-3)^ on the health of vulnerable populations | **1 = Always/often**  **2 = Sometimes**  **3 = Rarely**  **4 = Never**  **5 = Don't Know** | **Q10_2_2:**  **1 = Yes**  **2 = No** |
| **Q10_1_3** | Compare local data with state and national data related to the health of vulnerable populations | **1 = Always/often**  **2 = Sometimes**  **3 = Rarely**  **4 = Never**  **5 =** | **Q10_2_3:**  **1 = Yes**  **2 = No** |
| **Q10_1_4** | Evaluate the effectiveness of programs and activities to promote the health of vulnerable populations | **1 = Always/often**  **2 = Sometimes**  **3 = Rarely**  **4 = Never**  **5 = Don't Know** | **Q10_2_4:**  **1 = Yes**  **2 = No** |
| **Q10_1_5** | Track changes in local policy and built environments^[[4]](#footnote-4)^ that aim to improve the health of vulnerable populations | **1 = Always/often**  **2 = Sometimes**  **3 = Rarely**  **4 = Never**  **5 = Don't Know** | **Q10_2_5:**  **1 = Yes**  **2 = No** |
| **Q10_1_6** | Consider social and economic conditions in evaluating the effectiveness of interventions that promote the health of vulnerable populations | **1 = Always/often**  **2 = Sometimes**  **3 = Rarely**  **4 = Never**  **5 = Don't Know** | **Q10_2_6:**  **1 = Yes**  **2 = No** |
| **Q10_1_7** | Involve other community partners and local agencies/sectors in evaluations addressing the health of vulnerable populations | **1 = Always/often**  **2 = Sometimes**  **3 = Rarely**  **4 = Never**  **5 = Don't Know** | **Q10_2_7:**  **1 = Yes**  **2 = No** |
| **Q10_1_8** | Evaluate the effectiveness of collaborations developed to address the health needs of vulnerable populations | **1 = Always/often**  **2 = Sometimes**  **3 = Rarely**  **4 = Never**  **5 = Don't Know** | **Q10_2_8:**  **1 = Yes**  **2 = No** |
| **Q10_1_9** | Other (please specify): _______________ | **1 = Always/often**  **2 = Sometimes**  **3 = Rarely**  **4 = Never**  **5 = Don't Know** | **Q10_2_9:**  **1 = Yes**  **2 = No** |
| **Q10_1_9_TEXT** |  | **None** | **Q10_2_9_TEXT:**  **None** |

1. Which of the following ways does your LPHA leadership support work to promote the health of vulnerable populations?

Please choose either always/often, sometimes, rarely or never, and then indicate if you/your staff would like to gain skills or improve in this area.

| **Q11_1_1** | Prioritizes interventions to promote the health of vulnerable populations | **1 = Always/often**  **2 = Sometimes**  **3 = Rarely**  **4 = Never**  **5 = Don't Know** |
| --- | --- | --- |
| **Q11_1_2** | Supports ongoing training of workforce to develop skills needed to promote the health of vulnerable populations | **1 = Always/often**  **2 = Sometimes**  **3 = Rarely**  **4 = Never**  **5 = Don't Know** |
| **Q11_1_3** | Supports cultural competency training to enhance capacity to promote the health of vulnerable populations | **1 = Always/often**  **2 = Sometimes**  **3 = Rarely**  **4 = Never**  **5 = Don't Know** |
| **Q11_1_4** | Encourages the use of evidence^^[[5]](#footnote-5)^^ in making decisions to guide LPHA efforts to promote the health of vulnerable populations | **1 = Always/often**  **2 = Sometimes**  **3 = Rarely**  **4 = Never**  **5 = Don't Know** |
| Q11_1_12 | Supports “practice-academic”^^[[6]](#footnote-6)^^ partnerships and programs to enhance interventions to promote the health of vulnerable populations | **1 = Always/often**  **2 = Sometimes**  **3 = Rarely**  **4 = Never**  **5 = Don't Know** |
| **Q11_1_5** | Hires or appoints staff that are members of vulnerable populations | **1 = Always/often**  **2 = Sometimes**  **3 = Rarely**  **4 = Never**  **5 = Don't Know** |
| **Q11_1_6** | Hires or appoints staff with training to address the health of vulnerable populations | **1 = Always/often**  **2 = Sometimes**  **3 = Rarely**  **4 = Never**  **5 = Don't Know** |
| **Q11_1_7** | Fosters participation of staff when making important decisions regarding interventions to promote the health of vulnerable populations | **1 = Always/often**  **2 = Sometimes**  **3 = Rarely**  **4 = Never**  **5 = Don't Know** |
| **Q11_1_8** | Engages leadership from community partners^[[7]](#footnote-7)^ to promote the health of vulnerable populations | **1 = Always/often**  **2 = Sometimes**  **3 = Rarely**  **4 = Never**  **5 = Don't Know** |
| **Q11_1_9** | Educates the public and other stakeholders about the health of vulnerable populations | **1 = Always/often**  **2 = Sometimes**  **3 = Rarely**  **4 = Never**  **5 = Don't Know** |
| **Q11_1_10** | Coordinates with other LPHAs on initiatives related to promoting health in vulnerable populations | **1 = Always/often**  **2 = Sometimes**  **3 = Rarely**  **4 = Never**  **5 = Don't Know** |
| **Q11_1_11** | Coordinates with state, territorial or federal public health initiatives related to the health of vulnerable populations | **1 = Always/often**  **2 = Sometimes**  **3 = Rarely**  **4 = Never**  **5 = Don't Know** |
| **Q11_1_13** | Other (please specify): _______________ | **1 = Always/often**  **2 = Sometimes**  **3 = Rarely**  **4 = Never**  **5 = Don't Know** |
| **Q11_1_13_TEXT** |  | **None** |

1. **Q12.** Do you have a local champion that plays a leadership role in collaborative efforts to promote the health of vulnerable populations?

| Yes | **1** |
| --- | --- |
| No | **2** |
| Don’t Know | **3** |

If **YES** or **DK**,

6a. **Q13.** Please describe the skills and approaches that make the champion effective.

Describe here.

**Part II.** With this section of the survey we hope to gain some understanding of your LPHA’s climate with regard to implementing programs and policies that promote the health of vulnerable populations.

For each question, please rate how strongly you agree or disagree with the following statements.

*Note response options: Strongly disagree, Disagree, Neither, Agree, Strongly agree*

|  | Question/item | Response |
| --- | --- | --- |
| **Q14_1** | Programs and policies that promote the health of vulnerable populations are *important relative to other current initiatives within my organization*. | **1 = Strongly disagree**  **2 = Disagree**  **3 = Neither**  **4 = Agree**  **5 = Strongly agree** |
| **Q14_2** | Programs and policies that promote the health of vulnerable populations are *successfully prioritized among other competing priorities* in my organization. | **1 = Strongly disagree**  **2 = Disagree**  **3 = Neither**  **4 = Agree**  **5 = Strongly agree** |
| **Q14_3** | Programs and policies that promote the health of vulnerable populations *conflict/compete with other priorities* in my organization. | **1 = Strongly disagree**  **2 = Disagree**  **3 = Neither**  **4 = Agree**  **5 = Strongly agree** |

|  | Question/item | Response |
| --- | --- | --- |
| **Q15_1** | There is a strong *internal* push for my organization to promote programs and policies that improve the health of vulnerable populations. | **1 = Strongly disagree**  **2 = Disagree**  **3 = Neither**  **4 = Agree**  **5 = Strongly agree** |
| **Q15_2** | There is a strong *political* push for my organization to promote programs and policies that improve the health of vulnerable populations. | **1 = Strongly disagree**  **2 = Disagree**  **3 = Neither**  **4 = Agree**  **5 = Strongly agree** |
| **Q15_3** | There is a strong *community* push for my organization to promote programs and policies that improve the health of vulnerable populations. | **1 = Strongly disagree**  **2 = Disagree**  **3 = Neither**  **4 = Agree**  **5 = Strongly agree** |
| **Q15_4** | My organization *receives funding that requires* promoting programs and policies that improve the health of vulnerable populations. | **1 = Strongly disagree**  **2 = Disagree**  **3 = Neither**  **4 = Agree**  **5 = Strongly agree** |
| **Q15_5** | Programs and policies that improve the health of vulnerable populations are essential to meet the needs of the community served by my organization. | **1 = Strongly disagree**  **2 = Disagree**  **3 = Neither**  **4 = Agree**  **5 = Strongly agree** |

|  | Question/item | Response |
| --- | --- | --- |
| **Q16_1** | Programs and policies that promote the health of vulnerable populations fit well with the values and norms of *my organization*. | **1 = Strongly disagree**  **2 = Disagree**  **3 = Neither**  **4 = Agree**  **5 = Strongly agree** |
| **Q16_2** | Programs and policies that promote the health of vulnerable populations fit well with the values and norms of *other organizations that serve my community*. | **1 = Strongly disagree**  **2 = Disagree**  **3 = Neither**  **4 = Agree**  **5 = Strongly agree** |
| **Q16_3** | Programs and policies that promote the health of vulnerable populations fit well with *existing practices within my organization*. | **1 = Strongly disagree**  **2 = Disagree**  **3 = Neither**  **4 = Agree**  **5 = Strongly agree** |
| **Q16_4** | Programs and policies that promote the health of vulnerable populations would *complement a current program within my organization*. | **1 = Strongly disagree**  **2 = Disagree**  **3 = Neither**  **4 = Agree**  **5 = Strongly agree** |

|  | Question/item | Response |
| --- | --- | --- |
| **Q17_1** | Programs and policies that promote the health of vulnerable populations are well regarded by leadership in my organization | **1 = Strongly disagree**  **2 = Disagree**  **3 = Neither**  **4 = Agree**  **5 = Strongly agree** |
| **Q17_2** | Individuals/teams in my organization receive recognition for programs and policies that promote the health of vulnerable populations. | **1 = Strongly disagree**  **2 = Disagree**  **3 = Neither**  **4 = Agree**  **5 = Strongly agree** |
| **Q17_4** | Changes to goals are made based on feedback about the effectiveness of programs and policies to promote the health of vulnerable populations. | **1 = Strongly disagree**  **2 = Disagree**  **3 = Neither**  **4 = Agree**  **5 = Strongly agree** |
| **Q17_5** | Programs and policies to promote the health of vulnerable populations align with other goals of my organization (e.g., accreditation, continuous quality improvement) | **1 = Strongly disagree**  **2 = Disagree**  **3 = Neither**  **4 = Agree**  **5 = Strongly agree** |

**Part III.** In this section of the survey we would like to focus on obesity prevention through improving opportunities for health eating and physical activity as one example of a set of program and policy efforts to promote the health of vulnerable populations. *We recognize that while this may not be a primary focus area of your LPHA the information you provide in this section could help plan other approaches to addressing the needs of vulnerable populations in other program areas, as well.*

1. For each activity, please indicate the role that best fits your LPHA: leader, collaborator, or no role (check all that apply).

**Leader**: Your organization initiated and/or has led this effort

**Collaborator**: Your organization has supported others in this effort (e.g., provided assistance, data or information; met with coalitions and other organizations; contracted outside agencies; etc.)

**No role**: Your organization has served as neither leader nor collaborator in this effort

|  | Question/item | Response |
| --- | --- | --- |
|  | **In the past year, has your organization, either alone or with partners, been involved in promoting:** | **Checked = 1**  **Unchecked = “.”** |
| **Q20_1_1**  **-**  **Q20_1_4** | 1. Policies^^[[8]](#footnote-8)^^ or changes to the built environment^^[[9]](#footnote-9)^^ that improve access to healthier food choices in grocery stores, restaurants or food retailers for vulnerable populations | **…_1 = Leader**  **…_2 = Collaborator**  **…_3 = No role**  **…_4 = Don't Know** |
| **Q20_2_1**  **-**  **Q20_2_4** | 2. Policies and/or changes^^[[10]](#footnote-10)^^ that improve healthier food choices in schools, worksites and/or other local facilities^^[[11]](#footnote-11)^^ for vulnerable populations | **…_1 = Leader**  **…_2 = Collaborator**  **…_3 = No role**  **…_4 = Don't Know** |
| **Q20_3_1**  **-**  **Q20_3_4** | 3. Policies and/or changes^^[[12]](#footnote-12)^^ that improve healthier food choices through nutrition assistance programs like WIC or SNAP^^[[13]](#footnote-13)^^ | **…_1 = Leader**  **…_2 = Collaborator**  **…_3 = No role**  **…_4 = Don't Know** |
| **Q20_4_1**  **-**  **Q_20_4** | 4. Community media campaigns or health education interventions^^[[14]](#footnote-14)^^ to increase healthy food choices among vulnerable populations | **…_1 = Leader**  **…_2 = Collaborator**  **…_3 = No role**  **…_4 = Don't Know** |
| **Q20_5_1**  **-**  **Q20_5_4** | 5. Policies and/or changes to the built environment to increase opportunities for physical activity^^[[15]](#footnote-15)^^ for vulnerable populations | **…_1 = Leader**  **…_2 = Collaborator**  **…_3 = No role**  **…_4 = Don't Know** |
| **Q20_6_1**  **-**  **Q20_6_4** | 6. Policies and/or changes^^[[16]](#footnote-16)^^ to encourage physical activity in schools, worksites and/or other local facilities for vulnerable populations | **…_1 = Leader**  **…_2 = Collaborator**  **…_3 = No role**  **…_4 = Don't Know** |
| **Q20_7_1**  **-**  **Q20_7_4** | 7. Community media campaigns or health education interventions^^[[17]](#footnote-17)^^ to increase physical activity among vulnerable populations | **…_1 = Leader**  **…_2 = Collaborator**  **…_3 = No role**  **…_4 = Don't Know** |

1. **Q21.** Does your LPHA partner with other organizations^[[18]](#footnote-18)^ to promote the health of vulnerable populations through programs and policies that improve opportunities for healthy eating and physical activity? Please choose one.

| Yes | **1** |
| --- | --- |
| No | **2** |
| Don’t Know | **3** |

**If YES,**

2a. Please tell us if your LPHA is in partnership^[[19]](#footnote-19)^ with the following organizations by selecting all that apply.

|  |  | **Checked = 1**  **Unchecked = “.”** |
| --- | --- | --- |
| **Q22_1** | Elected officials in city or county government | **None** |
| **Q22_2** | Elected officials in state government | **None** |
| **Q22_3** | Local board of health | **None** |
| **Q22_4** | Schools | **None** |
| **Q22_5** | Childcare facilities | **None** |
| **Q22_6** | Hospitals | **None** |
| **Q22_7** | FQHC’s | **None** |
| **Q22_8** | Other community clinics | **None** |
| **Q22_9** | City, town, or county planning department | **None** |
| **Q22_10** | Parks and recreation department | **None** |
| **Q22_11** | Transportation department | **None** |
| **Q22_12** | Community-based organizations | **None** |
| **Q22_13** | Faith-based organizations | **None** |
| **Q22_22** | Cooperative extension | **None** |
| **Q22_14** | Agriculture department | **None** |
| **Q22_15** | Academic institutions | **None** |
| **Q22_16** | Business community | **None** |
| **Q22_17** | 4-H clubs or similar groups | **None** |
| **Q22_18** | Other (please specify): _______________ | **None** |
| **Q22_18_TEXT** | Other (please specify): _______________ | **None** |
| **Q22_19** | Other (please specify): _______________ | **None** |
| **Q22_19_TEXT** | Other (please specify): _______________ | **None** |

1. We realize that there are many factors that contribute to your LPHA’s work in promoting the health of vulnerable populations. We would like to ask about the things that may affect this work.

3a. Please list the top 1-3 things that help your LPHA promote the health of vulnerable populations.

**Q23_4_TEXT (RANK =1)** ____________________________________________________________

**Q23_1_TEXT(-2.)** _____________________________________________________________

**Q23_5_TEXT(-3.)** _____________________________________________________________

3b. Please list the top 1-3 things that get in the way of your LPHA promoting the health of vulnerable populations.

**Q24_1_TEXT(-1.)** _____________________________________________________________

**Q24_2_TEXT(-2.)** _____________________________________________________________

**Q24_3_TEXT(-3.)** _____________________________________________________________

**Part IV. Survey Respondent Information**

1. **Q25.** Though you may work in several capacities, which do you consider your primary position?

Please choose one.

| Agency Director | **1** |
| --- | --- |
| Health Officer | **2** |
| Division or Bureau Head/Division Deputy Officer | **3** |
| Department Head | **4** |
| Program Manager/Administrator/Coordinator | **5** |
| Health Educator | **6** |
| Environmental Health Specialist | **17** |
| Epidemiologist | **7** |
| Statistician | **8** |
| Health Policy Analyst | **9** |
| Program Planner | **10** |
| Program Evaluator | **11** |
| Community Health Nurse/PHN/RN | **12** |
| Dietician/Nutritionist | **13** |
| Academic Researcher | **14** |
| Academic Educator | **15** |
| Other (please specify) ____________________________ | **16** |

1. **Q26.** How long have you been in your current position? Please choose one.

| Less than 1 year | **1** |
| --- | --- |
| 1-3 years | **2** |
| - 1. years | **3** |
| 10 or more years | **4** |

1. **Q27.** How long have you worked for this agency/organization? Please choose one.

| Less than 1 year | **1** |
| --- | --- |
| 1-3 years | **2** |
| - 1. years | **3** |
| 10 or more years | **4** |

1. **Q28.** How long have you been involved in public health overall? Please choose one.

| Less than 1 year | **1** |
| --- | --- |
| 1-3 years | **2** |
| 4-9 years | **3** |
| 1. or more years | **4** |

**Incentive Information**

***IRB: For illustrative purposes, this information is listed in the same Word document. In the online survey, this information will be collected in a separate survey and database.**

1. As a thank you for completing our survey, we would like to offer you a gift card.

| Yes, I would like to receive a gift card. |  |
| --- | --- |
| No, I do not wish to receive a gift card or cannot receive an incentive due to my organization’s policy. |  |

**If YES**,

Please complete the following fields with your name, organization, telephone number, and email address so we can send you a gift card. This information will not be used for anything other than incentive purposes. Please note that names and contact information are kept in a separate database from survey responses.

Name: _____________________________________________________

Health Department: ______________________________________________________

Telephone Number: ___________________________________________

Email: ______________________________________________________

**If NO,**

Would you be willing to be a future contact in this study?

| Yes |  |
| --- | --- |
| No |  |

**If YES,**

Could you please include your name, organization, and email address so we can confirm that we have the correct contact information for you? Please note that names and contact information are kept in a separate database from survey responses.

Name: _____________________________________________________

Health Department: ______________________________________________________

Email: ___________________________________________

Thank you for your time and effort spent completing the survey. Your responses are invaluable to us.

Some participants will be randomly selected to complete the survey a second time. If you are one of those selected, we will contact you in the next 2 weeks.

**________________________________________________________________________________________**

**--End of Survey—**

1. [↑](#footnote-ref-1)
2. [↑](#footnote-ref-2)
3. In rural areas, this may be county level data; in metro areas, this could include census tracts or neighborhoods [↑](#footnote-ref-3)
4. Built environments refer to man-made settings (i.e., neighborhoods, buildings, parks, green space) and includes laws regarding zoning for those areas [↑](#footnote-ref-4)
5. Evidence includes The Community Guide, Community Health Assessment, US health surveillance, policy tracking and surveillance, systematic reviews, economic evaluation, and other publications. [↑](#footnote-ref-5)
6. Practice-academic partnership and programs can include practice-based research network, LPHA staff as adjunct professors, etc. [↑](#footnote-ref-6)
7. Could include other local agencies and/or non-public health sectors [↑](#footnote-ref-7)
8. May include formal laws, rules, and regulations as well as organizational guidelines (e.g., establishing healthy mobile markets, menu labeling in restaurants, and incentives for local distribution by farms) [↑](#footnote-ref-8)
9. ^10^Built environment refers to man-made settings (i.e. neighborhoods, buildings, parks, green space) and includes laws regarding zoning for those areas [↑](#footnote-ref-9)
10. e.g., offering free, safe drinking water, adoption of policies related to healthy meetings and vending, and ensuring standards for school lunches are met [↑](#footnote-ref-10)
11. Including after-school programs, recreation centers, child care facilities, churches, and/or local government agencies [↑](#footnote-ref-11)
12. e.g., encourage farmers markets to accept WIC, and grocery stores to improve WIC foods [↑](#footnote-ref-12)
13. Women, Infants, and Children (WIC), Supplemental Nutrition Assistance Program (SNAP, formerly the Food Stamp Program) and other assistance programs [↑](#footnote-ref-13)
14. e.g., promoting healthier foods at points-of-purchase, media campaigns to encourage breastfeeding, eliminating marketing of unhealthy food/drink near public places frequented by youth, and standards-based health education programs [↑](#footnote-ref-14)
15. e.g., Complete Streets, bike lanes, park upgrades, lighting improvements [↑](#footnote-ref-15)
16. e.g., improving access to bike equipment, increasing availability of open spaces, and supporting walk to school programs [↑](#footnote-ref-16)
17. e.g., point-of-decision prompts to encourage stair usage, standards-based health education programs, and worksite interventions with coaching/counseling [↑](#footnote-ref-17)
18. This may include, but is not limited to community based organizations, groups and key members of the community [↑](#footnote-ref-18)
19. e.g., participates in meetings, task forces, alliances, and/or coalitions [↑](#footnote-ref-19)
